# A Review of the Psyllid Genus *Epipsylla* (Hemiptera, Psyllidae) from the Chinese Mainland with Phylogenetic Considerations and the Description of a New Species †

**DOI:** 10.3390/insects16010099

**Published:** 2025-01-18

**Authors:** Zhixin He, Daniel Burckhardt, Xinyu Luo, Rongzhen Xu, Wanzhi Cai, Fan Song

**Affiliations:** 1 MOA Key Lab of Pest Monitoring and Green Management, Department of Entomology, College of Plant Protection, China Agricultural University, Beijing 100193, China; zhixinhee@126.com (Z.H.); ltquail@126.com (X.L.); 15738682275@163.com (R.X.); caiwz@cau.edu.cn (W.C.); 2 Naturhistorisches Museum, Augustinergasse 2, 4001 Basel, Switzerland; daniel.burckhardt@bs.ch

**Keywords:** Sternorrhancha, Psylloidea, taxonomy, Ciriacreminae, phylogeny, mitochondrial genome

## Abstract

*Epipsylla* Kuwayama, 1908, constitutes an Old World genus of psyllids with 15 described species. Based on characters of immatures, *Epipsylla* was recently assigned to Ciriacreminae (Psyllidae). The genus is morphologically well circumscribed but species are currently difficult to identify as many descriptions lack detail and precision. Eight species are reported from the Chinese mainland. Here, we provide diagnoses for the adults of these species and, as far as known, the fifth-instar immatures. Figures are provided of taxonomically relevant adult characters. A new species, *Epipsylla suni* sp. nov., is described from Yunnan (China). We provide illustrations of its habitus and morphological features, and list the host plant. Furthermore, we sequenced the mitochondrial genome of the new species and constructed a phylogenetic tree using thirteen protein-coding genes and two rRNA genes. The results of the molecular phylogenetic analysis using the maximum likelihood method support the assignment to Ciriacreminae.

## Simple Summary

The plant sap-sucking superfamily Psylloidea is characterized by its species with narrow host ranges. Fabaceae is the most important psyllid host family, mostly restricted to the family Psyllidae. *Epipsylla* is a distinctive genus of Psyllidae with 15 described species in the Old World, most of which develop on fabaceous hosts. Species identification in this genus is difficult as diagnostic species descriptions and accurate illustrations are lacking. Here, the eight species described from the Chinese mainland are reviewed, diagnoses and detailed illustrations of taxonomically relevant structures are provided, and a new species, *Epipsylla suni* sp. nov., is described from Yunnan (China). Based on a phylogenetic analysis of mitochondrial genome data, the phylogenetic status of *Epipsylla* in Ciriacreminae (Psyllidae), which was recently proposed, is supported.

## 1. Introduction

Jumping plant lice, also known as psyllids, constitute the superfamily Psylloidea, with over 4000 described species worldwide [1]. Characterized by narrow host ranges, they are mostly associated with eudicots and magnoliids, while hosts from the monocots and conifers are relatively rare. Furthermore, related psyllid species tend to develop on related host plants [2,3,4,5,6,7,8,9]. The most important host family in terms of associated psyllid genera is Fabaceae, which is almost exclusively restricted to Psyllidae, the most species-rich family of Psylloidea [8,10]. Seven of its nine named subfamilies recognized by Burckhardt et al. [11] contain species developing on Fabaceae, and many genera are completely or partly restricted to this host family. Ciriacreminae, for instance, consists of 19 genera and 182 described species. Eleven genera occur in the New World, six in the Old World, and only two in both. Twelve genera are restricted to hosts in Fabaceae, one genus each develops on Acanthaceae, Anacardiaceae, and Annonaceae, and two genera have hosts from several families, including Fabaceae, while for one genus, host information is lacking [11,12,13]. Modern taxonomic treatments are available for eight Neotropical genera (*Auchmerina*, *Auchmeriniella*, *Euceropsylla*, *Heteropsylla*, *Jataiba*, *Manapa*, *Mitrapsylla*, and *Queiroziella*) [10,11,14,15,16,17] and three Afrotropical genera (*Ciriacremum*, *Kleiniella*, and *Palmapenna*) [18,19], but they are lacking for the remaining eight genera. While six genera include less than 5 described species (*Caradocia*, *Geijerolyma*, *Hollisiana*. *Isogonoceraia*, *Telmapsylla*, and *Trigonon*), *Epipsylla* and *Insnesia* are more diverse with 15 and 21 recognized species, respectively [13]. Despite recent taxonomic treatments [12,20], the identification of *Epipsylla* species remains difficult as the existing descriptions are not diagnostic and lack accurate illustrations.

Kuwayama [21] erected *Epipsylla* for the two species from Taiwan, *E. albolineata* (type species), associated with *Mucuna macrocarpa* (Fabaceae), and *E. rubrofasciata*, associated with *Suregada aequorea* (Euphorbiaceae). Crawford [22] and Laing [23] each added a species from Southeast Asian and Oceania, respectively, though without host information. Yang and Li [24,25] and Yang [20] described thirteen species from Guangxi, Hainan, Taiwan, and Yunnan, developing mostly on Fabaceae with one on Malpighiaceae. Li et al. [12], finally, described a further species from Guangdong on *Millettia pachyloba* (Fabaceae) and synonymized three species, bringing the number of valid species to fifteen [12,13]. Unnamed *Epipsylla* species have also been reported from the Afrotropical and Australian biogeographical regions [7,12].

Kuwayama [21] ascribed *Epipsylla* to Psyllinae using exclusively forewing characteristics, a classification which was also followed by other authors (e.g., Yang [20]). Based on the shared presence of a terminal anus and a multi-layered circumanal ring in the fifth-instar larvae, White and Hodkinson [26] assigned *Epipsylla*, along with *Anomoneura*, to Anomoneurini (Psyllidae, Anomoneurinae). Li [27], who raised Psylloidea in rank to Psyllomorpha (an action which was not followed by subsequent authors), treated *Epipsylla* as being in Cornopsyllinae (Psylloidea, Euphaleridae) together with the genera *Cornopsylla*, *Epiacizzia*, and *Trisetipsylla*, supported by the presence of more than six metatibial spurs and antennae that are over 1.3 times as long as the head width. The presence of a posteriorly open crown of metatibial spurs in the adults and the lack of additional porefields in the immatures was used by Burckhardt and Ouvrard [28] to classify *Epipsylla* into Diaphorinini (Liviidae, Euphyllurinae). The extensive molecular phylogenetic analysis by Percy et al. [29] showed that the Euphyllurinae defined by Burckhardt and Ouvrard [28] is polyphyletic. Percy et al. [29] did not include *Epipsylla* in their molecular analysis. Burckhardt et al. [11] noted that the larvae of at least some *Epipsylla* species share sectasetae on the abdominal margin and a terminal anus with a multilayered circumanal ring with *Mitrapsylla*, and moved the genus to Ciriacreminae (Psyllidae). They further suggested that *Epipsylla*, *Caradocia*, and *Geijerolyma* constitute a putatively monophyletic group supported by the following characters long genal processes; very long antennae; metatibia lacking a genual spine; metatibial apical spurs forming a dense, posteriorly open crown; and metabasitarsus with two sclerotized spurs.

**Figure 1:**
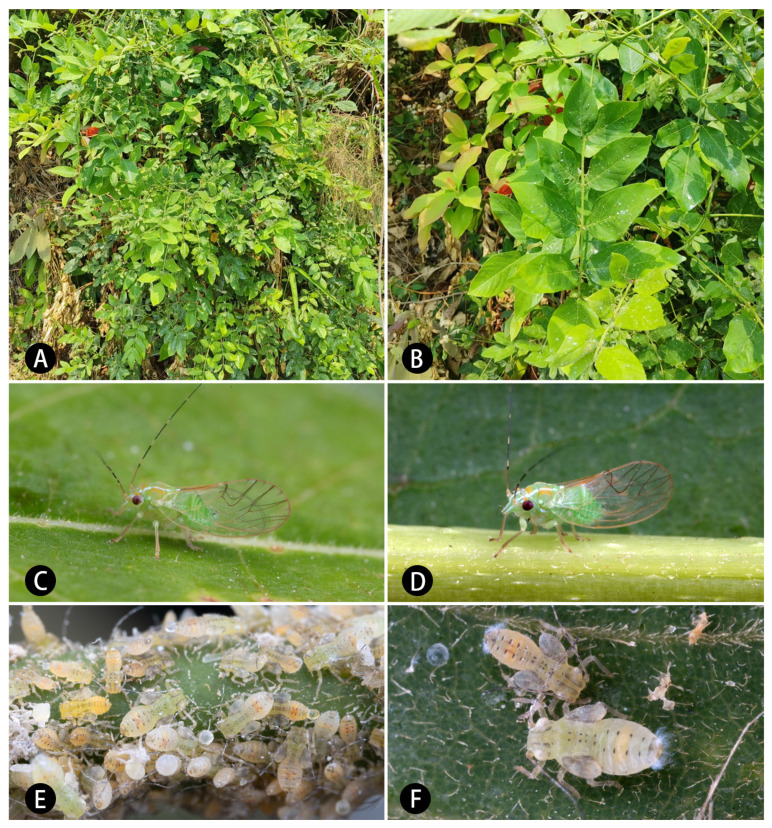
Habitus of *Epipsylla suni* sp. nov. (**A**,**B**) Host plant, *Derris taiwaniana*; (**C**,**D**) adults; (**E**,**F**) larvae.

Recently, an apparently undescribed *Epipsylla* species was discovered in Yunnan, necessitating a detailed review of the species occurring in the Chinese mainland (Figure 1). Here, we describe the new species and provide diagnoses for the species from the Chinese mainland, including illustrations of taxonomically relevant characters. Furthermore, we test the systematic position of *Epipsylla* in Ciriacreminae with the use of molecular characters.

## 2. Materials and Methods

### 2.1. Morphological Study

The bulk of the material is deposited in the Entomological Museum, China Agricultural University, Beijing, China (CAU), with additional specimens in the Institute of Entomology, Guizhou University (IEGU); the National Zoological Museum of China, Institute of Zoology, Chinese Academy of Sciences, Beijing, China (NZMC); the Natural History Museum, London, UK (BMNH); and the Naturhistorisches Museum, Basel, Switzerland (NHMB).

**Figure 2:**
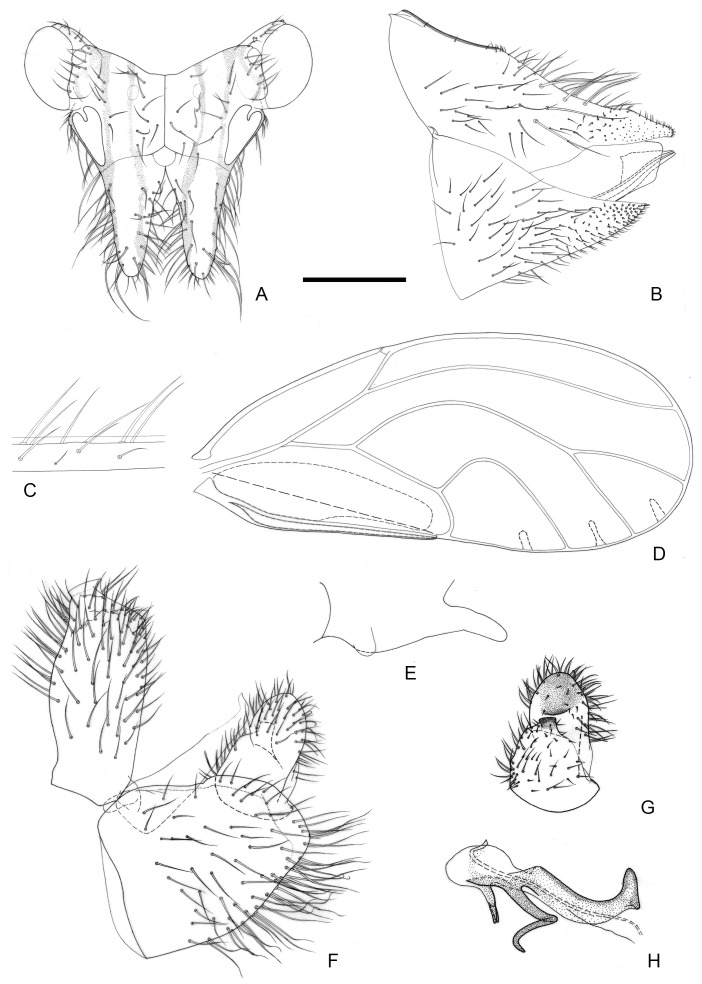
*Epipsylla crotalariae* Yang & Li, 1984. (**A**) Head (antennae removed); (**B**) female terminalia in lateral view; (**C**) setae on vein C + Sc; (**D**) forewing; (**E**) meracanthus; (**F**) male terminalia in lateral view (ignoring distal segment of aedeagus and phallobase); (**G**) inner surface of paramere; (**H**) distal segment of aedeagus. Scale bar (mm): (**A**) 0.3; (**B**) 0.15; (**C**) 0.1; (**D**) 0.6; (**E**) 0.15; (**F**) 0.15; (**G**) 0.1; (**H**) 0.1.

**Figure 3:**
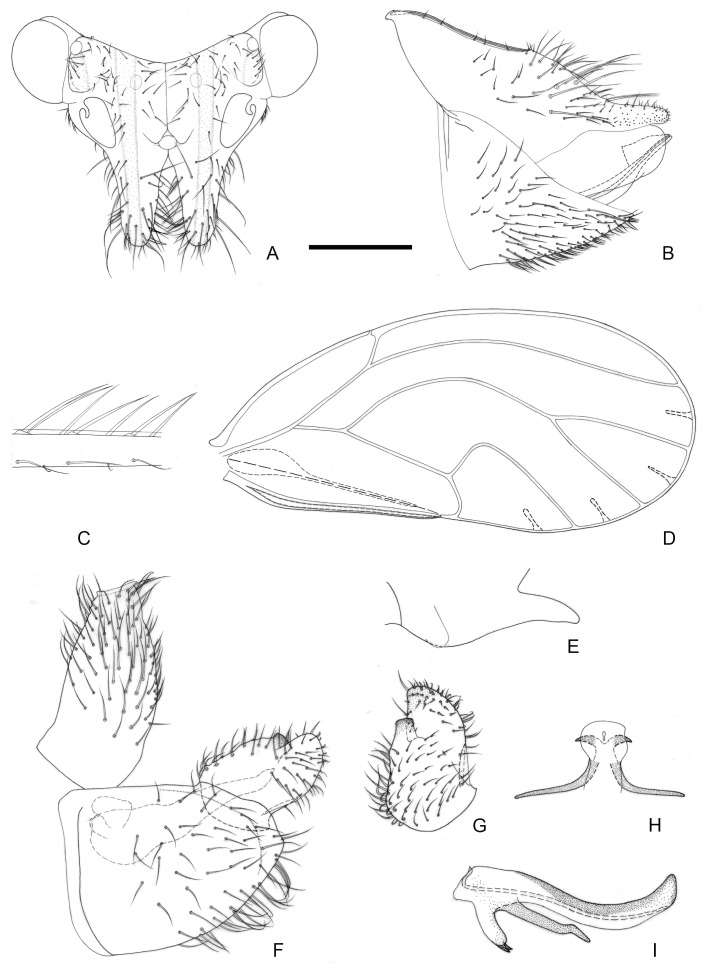
*Epipsylla guangxiana* Yang & Li, 1983. (**A**) Head (antennae removed); (**B**) female terminalia in lateral view; (**C**) setae on vein C + Sc; (**D**) forewing; (**E**) meracanthus; (**F**) male terminalia in lateral view (ignoring distal segment of aedeagus and phallobase); (**G**) inner surface of paramere; (**H**) distal segment of aedeagus in dorsal view; (**I**) distal segment of aedeagus in lateral view. Scale bar (mm): (**A**) 0.25; (**B**) 0.2; (**C**) 0.1; (**D**) 0.5; (**E**) 0.15; (**F**) 0.1; (**G**) 0.1; (**H**) 0.1; (**I**) 0.1.

**Figure 4:**
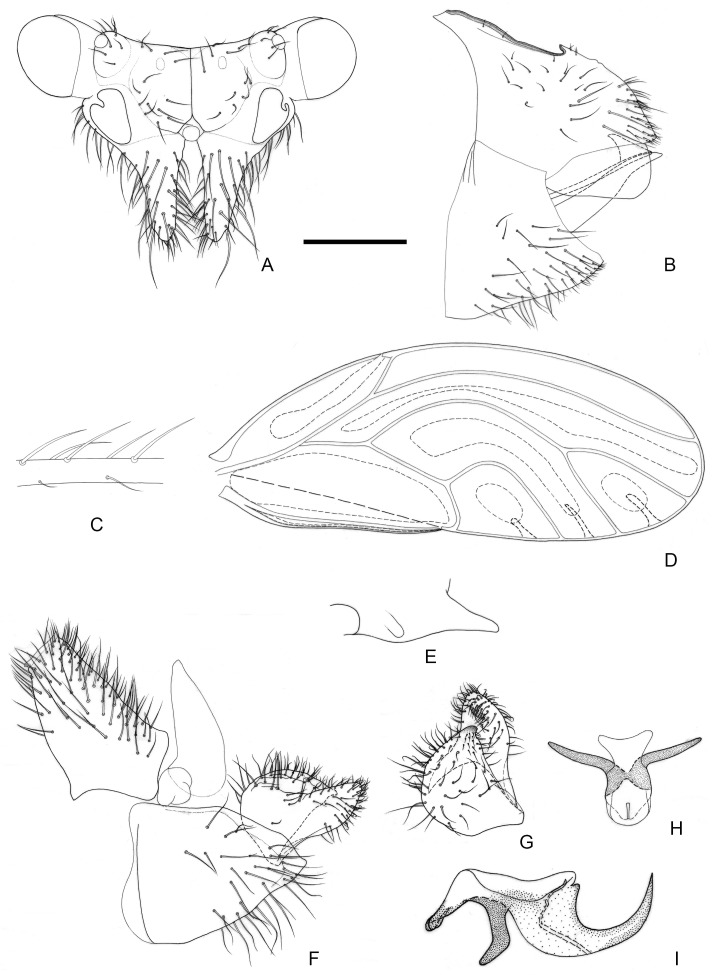
*Epipsylla hainanana* Yang & Li, 1983. (**A**) Head (antennae removed); (**B**) female terminalia in lateral view; (**C**) setae on vein C + Sc; (**D**) forewing; (**E**) meracanthus; (**F**) male terminalia in lateral view (ignoring distal segment of aedeagus and phallobase); (**G**) inner surface of paramere; (**H**) distal segment of aedeagus in dorsal view; (**I**) distal segment of aedeagus in lateral view. Scale bar (mm): (**A**) 0.25; (**B**) 0.25; (**C**) 0.1; (**D**) 0.5; (**E**) 0.15; (**F**) 0.12; (**G**) 0.1; (**H**) 0.15; (**I**) 0.1.

**Figure 5:**
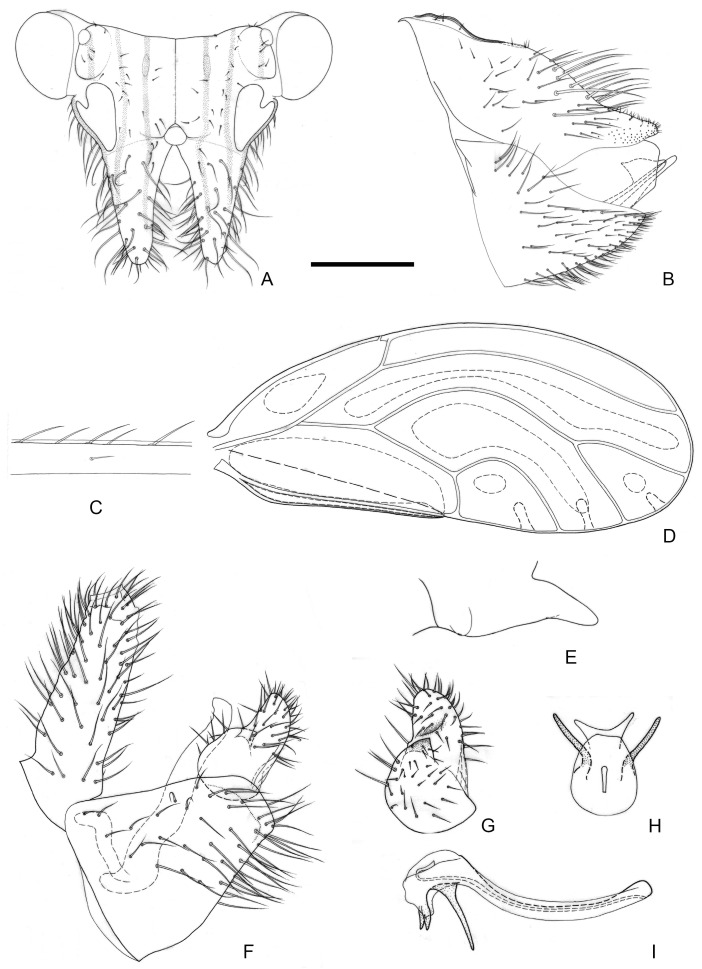
*Epipsylla liui* Yang & Li, 1983. (**A**) Head (antennae removed); (**B**) female terminalia in lateral view; (**C**) setae on vein C + Sc; (**D**) forewing; (**E**) meracanthus; (**F**) male terminalia in lateral view (ignoring distal segment of aedeagus and phallobase); (**G**) inner surface of paramere; (**H**) distal segment of aedeagus in dorsal view; (**I**) distal segment of aedeagus in lateral view. Scale bar (mm): (**A**) 0.25; (**B**) 0.2; (**C**) 0.1; (**D**) 0.5; (**E**) 0.15; (**F**) 0.12; (**G**) 0.1; (**H**) 0.2; (**I**) 0.1.

**Figure 6:**
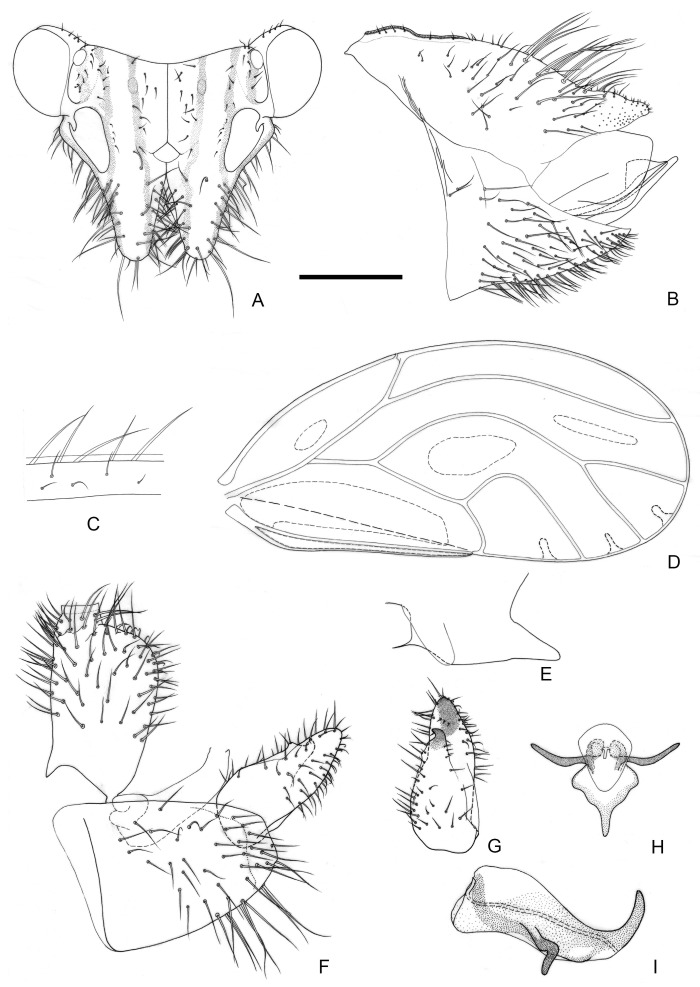
*Epipsylla millettiae* Li, Yang & Burckhardt, 2015. (**A**) Head (antennae removed); (**B**) female terminalia in lateral view; (**C**) setae on vein C + Sc; (**D**) forewing; (**E**) meracanthus; (**F**) male terminalia in lateral view (ignoring distal segment of aedeagus and phallobase); (**G**) inner surface of paramere; (**H**) distal segment of aedeagus in dorsal view; (**I**) distal segment of aedeagus in lateral view. Scale bar (mm): (**A**) 0.25; (**B**) 0.2; (**C**) 0.1; (**D**) 0.5; (**E**) 0.15; (**F**) 0.12; (**G**) 0.1; (**H**) 0.15; (**I**) 0.1.

**Figure 7:**
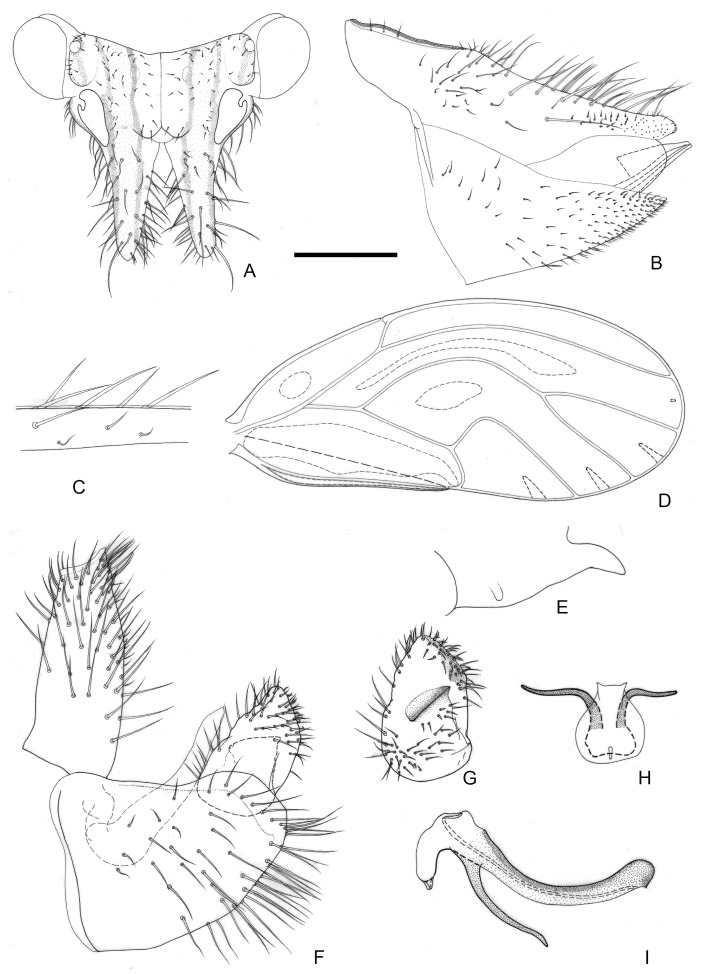
*Epipsylla mucunae* Yang & Li, 1984. (**A**) Head (antennae removed); (**B**) female terminalia in lateral view; (**C**) setae on vein C + Sc; (**D**) forewing; (**E**) meracanthus; (**F**) male terminalia in lateral view (ignoring distal segment of aedeagus and phallobase); (**G**) inner surface of paramere; (**H**) distal segment of aedeagus in dorsal view; (**I**) distal segment of aedeagus in lateral view. Scale bar (mm): (**A**) 0.25; (**B**) 0.15; (**C**) 0.1; (**D**) 0.6; (**E**) 0.15; (**F**) 0.15; (**G**) 0.1; (**H**) 0.15; (**I**) 0.1.

**Figure 8:**
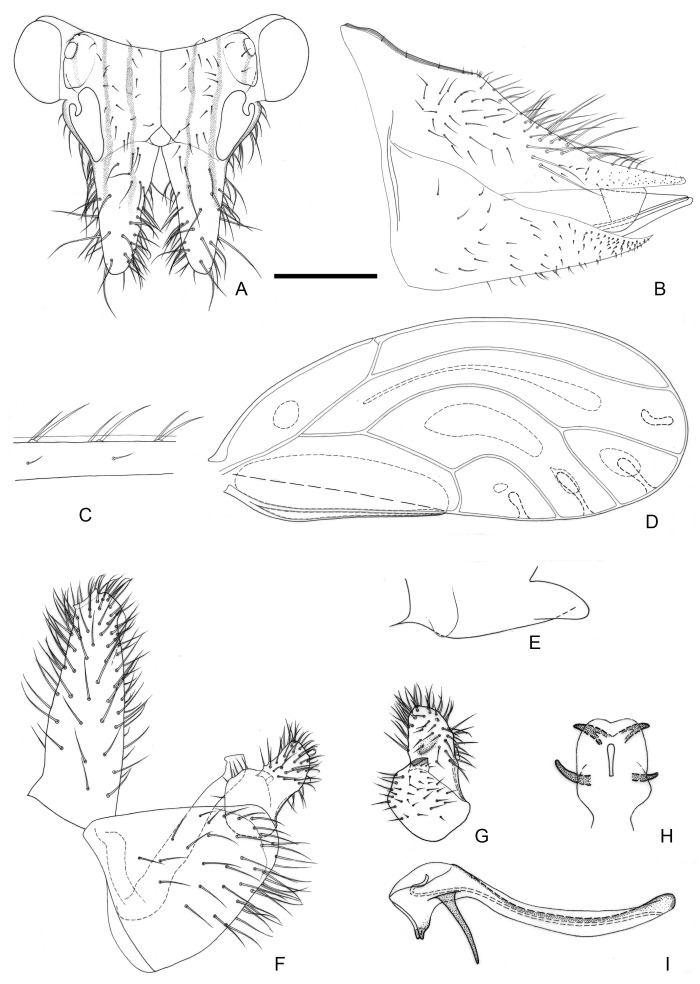
*Epipsylla nadana* Yang & Li, 1983. (**A**) Head (antennae removed); (**B**) female terminalia in lateral view; (**C**) setae on vein C + Sc; (**D**) forewing; (**E**) meracanthus; (**F**) male terminalia in lateral view (ignoring distal segment of aedeagus and phallobase); (**G**) inner surface of paramere; (**H**) distal segment of aedeagus in dorsal view; (**I**) distal segment of aedeagus in lateral view. Scale bar (mm): (**A**) 0.25; (**B**) 0.15; (**C**) 0.1; (**D**) 0.5; (**E**) 0.15; (**F**) 0.12; (**G**) 0.12; (**H**) 0.2; (**I**) 0.1.

**Figure 9:**
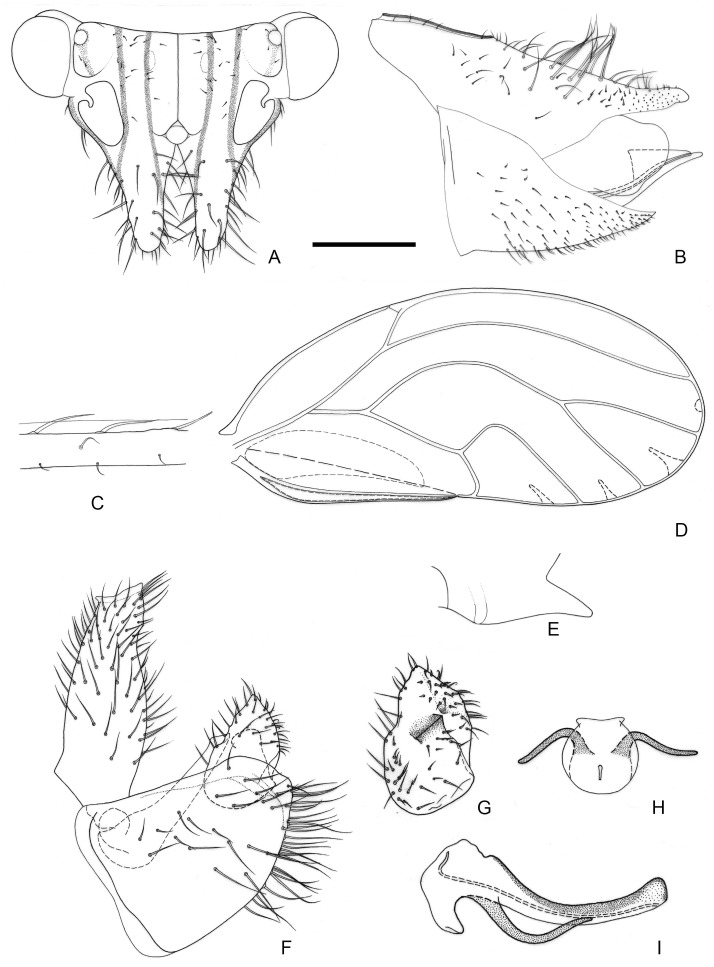
*Epipsylla puerariae* Yang & Li, 1983. (**A**) Head (antennae removed); (**B**) female terminalia in lateral view; (**C**) setae on vein C + Sc; (**D**) forewing; (**E**) meracanthus; (**F**) male terminalia in lateral view (ignoring distal segment of aedeagus and phallobase); (**G**) inner surface of paramere; (**H**) distal segment of aedeagus in dorsal view; (**I**) distal segment of aedeagus in lateral view. Scale bar (mm): (**A**) 0.2; (**B**) 0.15; (**C**) 0.1; (**D**) 0.5; (**E**) 0.18; (**F**) 0.15; (**G**) 0.1; (**H**) 0.12; (**I**) 0.1.

**Figure 10:**
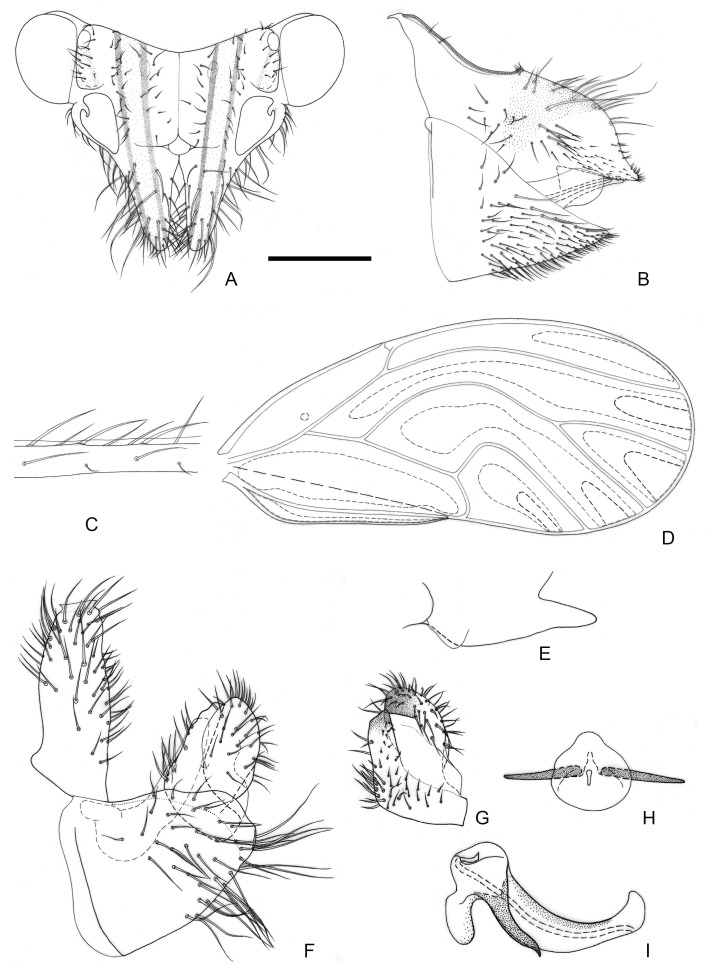
*Epipsylla suni* Luo, Burckhardt & Cai, sp. nov. (**A**) Head (antennae removed); (**B**) female terminalia in lateral view; (**C**) setae on vein C + Sc; (**D**) forewing; (**E**) meracanthus; (**F**) male terminalia in lateral view (ignoring distal segment of aedeagus and phallobase); (**G**) inner surface of paramere; (**H**) distal segment of aedeagus in dorsal view; (**I**) distal segment of aedeagus in lateral view. Scale bar (mm): (**A**) 0.25; (**B**) 0.2; (**C**) 0.1; (**D**) 0.5; (**E**) 0.15; (**F**) 0.15; (**G**) 0.12; (**H**) 0.12; (**I**) 0.1.

The preparation of slides was as follows: whole insects were boiled in potassium hydroxide (KOH) solution for ten minutes, naturally cooled down after heating stopped, then washed in distilled water, and finally mounted on a slide in glycerine. All examinations were made and photos taken with an Olympus^®^ BX41 microscope (Olympus, Tokyo, Japan). In the forewing drawings (Figure 2, Figure 3, Figure 4, Figure 5, Figure 6, Figure 7, Figure 8, Figure 9 and Figure 10D), the thin dashed lines represent the distribution of the fields of surface spinules (on the dorsal surface), while the thick dashed lines represent the extent of the fields of radular spinules (on the ventral surface).

Measurements were taken from slide-mounted specimens. For adults, BL = total body length measured from the anterior margin of the vertex to the tip of the folded forewings, HW = head width, AL = antennal length, TW = mesoscutum width, WL = forewing length, and TL = metatibial length. For fifth-instar immature, BL = total body length, HW = head width, AL = antennal length, and FL = maximum forewing pad length.

The morphological terminology mainly follows Bastin et al. [30], and the nomenclature of plants accords with POWO [31]. The concept of host plant is that of Burckhardt et al. [5].

### 2.2. DNA Extraction

The genomic DNA was extracted using a Dneasy Blood and Tissue Kit (Qiagen, Beijing, China) following the manufacturer’s protocol.

### 2.3. Mitogenome Sequencing, Assembly, and Annotation

The *COX1* fragment (~610 bp) was amplified by polymerase chain reaction (PCR) with newly designed primers UCOIF (50-TTTCHACNAACCATAAGGAYATTGG-30) and UCOIR (50-TANACTTCTGGGTGTCCAAAAAATCA-30). Short PCR amplifications were carried out using Qiagen Taq DNA polymerase (Qiagen, Beijing, China) with the following cycling conditions: 5 min at 94 °C, followed by 35 cycles of 50 s at 94 °C, 50 s at 48–55 °C, and 1–2 min at 72 °C. The final elongation step was continued for 10 min at 72 °C. The PCR products were analyzed by 1.0% agarose gel electrophoresis and then sequenced by Sanger sequencing at Sangon Biotech (Beijing, China).

Library setting and sequencing were done by the Beijing Genomics institution (Beijing, China). The BGISEQ-500 library was prepared using genomic DNA with an average insert size of 200–400 bp, and pair-end sequencing with 150 bp lengths was performed using the BGISEQ-500 sequencer with the processed libraries. For each library, 4 Gb of clean data were obtained after removing reads containing adaptor contamination poly-Ns (>15 bp Ns) or >75 bp bases with quality score ≤ 3. Clean reads were used in de novo assembly by using IDBA-UD [32], with minimum and maximum k-values of 41 and 141 bp, respectively. To identify the corresponding mitogenome assemblies, the assembled contigs were searched with the *COX1* sequence using BLAST with at least 98% similarity. To investigate the accuracy of the assembly, clean reads were mapped onto the obtained mitogenome sequences using Geneious prime 2022.2.2 (http://www.geneious.com/ accessed on 20 August 2024). The mitochondrial genome was extracted and annotated using MitoZ [33]. The annotation accuracy of 13 protein coding genes and 2 rRNA genes was examined by Geneious prime. The online tRNAscan-SE service (http://lowelab.ucsc.edu/tRNAscan-SE/ accessed on 20 August 2024) was used to confirm the locations of the tRNA genes. The complete mitogenome has been uploaded to GenBank (Table 1).

### 2.4. Phylogenetic Analysis

Combining the newly sequenced mitogenome and sequences from GenBank, a total of 17 species from Psyllidae and 1 from Triozidae were included in our phylogenetic analysis. *Trioza urticae* (Triozidae) was selected as an outgroup (Table 1).

The two rRNA genes of each species were separately aligned using the L-INS-I strategy in the MAFFT algorithm [34] and trimmed using trimAl v1.2 [35]. The 13 PCGs of each species were translated into amino acids and then aligned using the MAFFT algorithm individually. The sequences of amino acids were trimmed and back-translated based on the original DNA sequences in trimAl v1.2. Thirteen PCGs and two rRNA genes were concatenated using FASconCAT-G v 1.04 [36], while the partition file was obtained.

Phylogenetic trees constructed using the maximum likelihood (ML) method were implemented in the IQ-TREE web server with a dataset partitioned by genes [37]. The robustness of the tree topology was further assessed by SH-aLRT, and 1000 ultrafast bootstrap replicates were implemented.

## 3. Results

### 3.1. Taxonomy

*Epipsylla* Kuwayama, 1908.
*Epipsylla* Kuwayama [21]: 178.
Type species: *Epipsylla albolineata* Kuwayama, by original designation.
Diagnoses of adults and last-instar immatures by Li et al. [12].

#### 3.1.1. *Epipsylla crotalariae* Yang & Li, 1984

Figure 2 and Figure 12A,B.

*Epipsylla cortalariae* Yang & Li [25]: 251; Yang & Li [38] (citation); Hodkinson [39] (list); Li [27] (redescription).

Material examined: Holotype: male, China, Yunnan, Longchuan, Husa, 24°46′72″ N, 97°89′32″ E, alt. 1430 m, 29 April 1981, Fasheng Li leg. on *Crotalaria retusa*; dry-mounted; CAU. Paratype: one female, same information as holotype.

Diagnosis: Adult. Body dark green. Head bearing 1 + 1 white longitudinal stripe on both sides, surrounding lateral ocelli and extended to postocular sclerite, with black edge inside, black side mixed with partial outside black edge of middle stripe (Figure 2A). Genal processes thick and long, apex rounded and blunt, bearing setae (Figure 2A). Metatibia without basal spine, apex with an open, dense crown of 7–8 strongly sclerotized apical spurs. Forewing oval, widest in the middle; pterostigma very small (Figure 2D); fore margin with conspicuous dark long setae, gradually getting shorter toward wing apex (Figure 2C); surface spinules absent except for cell cu_2_, radular spinules forming narrow stripes in the middle of cells m1, m2, and cu1 along wing margin (Figure 2D). Meracanthus slender, uniform thickness, apex rounded and blunt (Figure 2E). Male terminalia: Proctiger 1.7 times as long as paramere (Figure 2F). Paramere as in Figure 2G; outer lobe evenly rounded apically, with sclerotized area on inner surface extended, covering apical third almost entirely; inner lobe short, apex strongly sclerotized, angular, extending to distal third of outer lobe. Basal segment of aedeagus almost entirely straight, gradually narrowing to apex which is thin and curved (Figure 2F). Distal segment of aedeagus as in Figure 2H; 1.2 times as long as basal segment; base forming short, subacute dorsal horn; ventro-lateral sclerotized processes almost in apical third, long, tortile; ventral process subapically, divided in apical half; apical dilatation sub-oval; sclerotized end tube near aedeagal apex. Female terminalia: Overall high and long. Dorsal part of apical half of proctiger strongly raised, and apex bearing a tuft of fine setae (Figure 2B).

Measurements (mm): Male (*n* = 1): BL 4.38; AL 4.48; HW 0.87; TW 0.87; WL 3.37. Female (*n* = 1): BL 4.77; AL 4.78; HW 0.97; TW 0.88; WL 3.63 (from Li 2011).

Fifth-instar immature unknown.

Host plant: Adults were collected on *Crotalaria retusa* L. (Fabaceae), which is a possible host.

Distribution: China (Yunnan).

#### 3.1.2. *Epipsylla guangxiana* Yang & Li, 1983

Figure 3 and Figure 12C,D.

*Epipsylla guangxiana* Yang & Li [24]: 314; Hodkinson [39] (list); Li [27] (description).

*Epipsylla whitfordiodendritis* Yang & Li [24]: 315; Hodkinson [39] (list); Li et al. [12] (synonymy).

Material examined: Holotype: holotype of *E. guangxiana*: male, China, Guangxi, Tianlin, Langping, 24°48′45″ N, 106°36′54″ E, alt. 1500 m, 29 May 1982, Fasheng Li & Ceekun Yang leg. on *Padbruggea filipes*; dry-mounted; CAU; holotype of *E. whitfordiodendritis*: same information as *E. guangxiana*. Paratypes: paratypes of *E. guangxiana*: four females (dry-mounted) and two immatures (in 75% ethanol), same information as holotype; paratypes of *E. whitfordiodendritis*: eight males, ten females, same information as holotype.

Diagnosis: Adult. Body green. Vertex bearing 1 + 1 white stripe with black edge in middle, extending forward but not reaching apex of genal processes; head bearing 1 + 1 white longitudinal stripe on both sides, with black edge inside. (Figure 3A). Genal processes thick and long, apex rounded and blunt, bearing slender setae (Figure 3A). Metatibia without basal spine, apex with an open, dense crown of eight strongly sclerotized apical spurs. Forewing bearing three rows (dorsal surface, ventral surface, and anterior margin) of long setae in vein C + Sc (Figure 3C); surface spinules absent apart from base of cell cu_2_, radular spinules forming narrow stripes in the middle of cells r_2_, m_1_, m_2_ and cu_1_ along wing margin (Figure 3D). Meracanthus slender, apex somewhat acute, overall decurved (Figure 3E).

Male terminalia: Proctiger 1.4–1.5 times as long as paramere (Figure 3F). Paramere as in Figure 3G; outer lobe irregularly rounded apically, with sclerotized area on inner surface restricted to area near apex; inner lobe short, apex strongly sclerotized, angular, extending to distal quarter of outer lobe. Basal segment of aedeagus weakly sinuous, gradually narrowing to apex (Figure 3F). Distal segment of aedeagus as in Figure 3H,I; 1.2 times as long as basal segment; base forming short, blunt dorsal tubercle; ventro-lateral sclerotized processes in apical quarter, moderately long, irregularly weakly curved; ventral process subapically, divided in apical quarter; apex not dilated; sclerotized end tube near aedeagal apex. Female terminalia: Proctiger narrower in apical half, apex rounded and blunt, with sparse tiny setae (Figure 3B).

Measurements (mm): Male (*n* = 3); BL 3.76–3.90; AL 4.85–4.88; HW 0.85–0.86; TW 0.70–0.71; WL 3.01–3.12. Female (*n* = 3): BL 4.13–4.27; AL 5.03–5.54; HW 0.89–0.90; TW 0.72–0.75; WL 3.33–3.34.

Fifth-instar *immature: Abdominal margin bearing a pair of sectasetae. Outer circumanal ring comprising more than six rows of oval pores laterally which, between the anterior and posterior margins, consist of subrectangular pores.

Host plant: *Padbruggea filipes* (Dunn) Craib (Fabaceae).

Distribution: China (Guangxi).

#### 3.1.3. *Epipsylla hainanana* Yang & Li, 1983

Figure 4 and Figure 12E,F.

*Epipsylla hainanana* Yang & Li [24]: 308; Hodkinson [39] (list); Li [40] (list); Li [27] (redescription). Li et al. [12] (redescription).

Material examined: Holotype: male, China, Hainan, Lingshui, Diaoluoshan, Nanxi, 18°70′31″ N, 109°83′88″ E, 27 April 1965, Sikong Liu leg. dry-mounted; CAU. Paratypes: six males, seven females, same information as holotype.

Diagnosis: Adult. Body greyish-green. Head without longitudinal stripes (Figure 4A). Mesopraescutum dark green in anterior half, separated from the background area by a black edge; mesoscutum bearing 2 + 2 dark green stripes with black edge on both sides. Genal processes slightly longer than vertex, base separated, apex more acute with dense long setae (Figure 4A). Metatibia without basal spine, apex with an open, dense crown of seven strongly sclerotized apical spurs. Forewing elongated in shape, bearing three rows (dorsal surface, ventral surface, and anterior margin) of long setae in anterior margin (Figure 4C); range of surface spinules relatively large and radular spinules slender, degenerated in r2 (Figure 4D). Meracanthus tapered evenly toward apex, apex somewhat acute (Figure 4E). Male terminalia: Proctiger 1.4–1.5 times as long as paramere (Figure 4F). Paramere as in Figure 4G; outer lobe irregularly triangular, blunt apically, with sclerotized area on inner surface forming transversely oblique ridge in distance from margin; inner lobe large, triangular, sclerotized apex, angular, extending to distal quarter of outer lobe. Basal segment of aedeagus almost straight, gradually narrowing from broad base to narrow apex (Figure 4F). Distal segment of aedeagus as in Figure 4H,I; 1.3 times as long as basal segment; base forming long, falci-form pointed process; ventro-lateral sclerotized processes in apical third, short, irregularly curved, blunt; ventral process apical, not divided into apical processes; apex not dilated; sclerotized end tube proximal of aedeagal middle. Female terminalia: Whole short and tall. Dorsal surface of proctiger plumped up, with a row of long setae (Figure 4B).

Fifth-instar immature and host plant unknown.

Distribution: China (Hainan).

#### 3.1.4. *Epipsylla liui* Yang & Li, 1983

Figure 5.

*Epipsylla liui* Yang & Li [24]: 309; Hodkinson [39] (list); Li [40] (list); Li [27] (redescription).

Material examined: Paratypes: three males, two females, Hainan, Lingshui, Diaoluoshan, Nanxi, 18°69′78″ N, 109°84′69″ E, 27 April 1965, Sikong Liu leg. dry-mounted; CAU.

Diagnosis: Adult. Body yellowish brown. Vertex bearing 1 + 1 white stripe with black edge in middle part, slightly wider in genal processes, extended forward but not reaching apex of genal processes; head bearing 1 + 1 short white longitudinal stripe on both sides, with black edge inside, black edge surrounding most of lateral ocelli; external margin of antennal insertion bearing a black edge, extended to basal two-fifths of genal processes, not mixed with middle longitudinal stripe; another black stripe on posterior base of genal processes, extending to about two-fifths of genal processes (Figure 5A). Genal processes slightly longer than vertex, whole curved inward, setae short (Figure 5A). Metatibia without basal spine, apex with an open, dense crown of seven strongly sclerotized apical spurs. Forewing bearing relatively short and sparse setae at anterior margin (Figure 5C); range of somewhat large surface spinules and relatively wide and short radular spinules, that of r_2_ disappeared (Figure 5D). Meracanthus decurved, apex somewhat acute (Figure 5E).

Male terminalia: Proctiger 2 times as long as paramere (Figure 5F). Paramere as in Figure 5G; outer lobe irregularly rounded apically, with sclerotized area on inner surface forming oblique ridge in apical third of paramere; inner lobe short, apex strongly sclerotized, pointed, extending to the middle of outer lobe. Basal segment of aedeagus weakly sinuous, only weakly narrowing to apex (Figure 5F). Distal segment of aedeagus as in Figure 5H,I; 1.1 times as long as basal segment; base forming at most inconspicuous dorsal tubercle; ventro-lateral sclerotized processes subapical, relatively short and almost straight; ventral process subapically, divided in the middle; apex weakly dilated; sclerotized end tube near aedeagal apex. Female terminalia: Whole taller than long. Proctiger rounded and plump in basal half, apical expanding area short (Figure 5B).

Fifth-instar immature and host plant unknown.

Distribution: China (Hainan).

#### 3.1.5. *Epipsylla millettiae* Li, Yang & Burckhardt, 2015

Figure 6 and Figure 12G,H.

*Epipsylla millettiae* Li et al. [12]: 137.

Material examined. Paratypes: five males, five females, China, Guangdong, Zhanjiang, Southern Aisa Subtropical Botanical Garden, 21°16′31″ N, 110°27′63″ E; 19 April 2013, Bin Li & Meng Jiao leg. on *Millettia pachyloba*; dry-mounted; IEGU.

Diagnosis: Adult. Body green. Head bearing 1 + 1 light greenish yellow longitudinal stripes with black edge in middle part, extended forward and not reaching genal processes; head bearing 1 + 1 white longitudinal stripes on both sides, with black edge on inner margin; a narrow black band extended forward along outer edge of antennal insertion on genal side, retracting back to middle of genal processes (Figure 6A). Genal processes stout, apex rounded and blunt, densely elongated setae (Figure 6A). Metatibia without basal spine, apex with an open, dense crown of 7–8 strongly sclerotized apical spurs. Forewing bearing two rows (anterior margin and ventral surface) of long seta along vein C + Sc (Figure 6C); range of surface spinules small and radular spinules degenerated in r2 (Figure 6D). Meracanthus relatively slender, apex somewhat acute, whole slightly curved downward (Figure 6E). Male terminalia: Proctiger 1.1 times as long as paramere (Figure 6F). Paramere as in Figure 6G; outer lobe irregularly, narrowly triangular, blunt apically, with sclerotized area on inner surface covering area near apex; inner lobe long, apex strongly sclerotized, forming anteriorly directed hook, extending to apical quarter of outer lobe. Basal segment of aedeagus mostly straight, slightly curved basally and apically, relatively evenly narrowing to apex (Figure 6F). Distal segment of aedeagus as in Figure 6H,I; 1.2 times as long as basal segment; base forming slightly curved, long dorsal process; ventro-lateral sclerotized processes in the middle, relatively short and curved; ventral process apical, not divided distally; apex broad, truncated; sclerotized end tube at aedeagal apex. Female terminalia: Dorsal outline of proctiger, in lateral view, weakly sinuate; apical expanded area short, dorsal surface plump, apex rounded and blunt, with sparse fine setae (Figure 6B).

Measurements (mm): Male (*n* = 4): BL 3.00–3.55; AL 3.59–3.82; HW 0.72–0.78; TW 0.59–0.71; WL 2.39–2.71. Female (*n* = 3): BL 3.28–3.73; AL 3.62–3.94; HW 0.73–0.78; TW 0.63–0.73; WL 2.56–2.92.

Fifth-instar immature unknown.

Host plant: Adults were collected on *Millettia pachyloba* Drake (Fabaceae), which is a likely host.

Distribution: China (Guangdong).

#### 3.1.6. *Epipsylla mucunae* Yang & Li, 1984

Figure 7 and Figure 13A,B.

*Epipsylla mucunae* Yang & Li [25]: 253; Hodkinson [39] (list); Yang & Li [38] (list); Li [27] (redescription).

*Epipsylla ruiliana* Yang & Li [25]: 25; Hodkinson [39] (list); Li et al. [12] (synonym).

*Epipsylla yunnanica* Yang & Li [25]: 255; Hodkinson [39] (list); Li et al. [12] (synonym).

Material examined: Holotype: *E. mucunae*: male, China, Yunnan, Ruili, Guangshuang, alt. 750 m, 23°94′38″ N, 97°78′11″ E, 1 May 1981, Fasheng Li leg. on *Mucuna macrocarpa*; dry-mounted; CAU; holotype of *E. ruiliana*: same information as *E. mucunae*; holotype of *E. yunnanica*: same information as *E. mucunae*. Paratypes: paratypes of *E. mucunae*: four males, same information as holotype; paratypes of *E. ruiliana*: three males, two females, same information as holotype; paratypes of *E. yunnanica*: four males, nine females, same information as holotype.

Diagnosis: Adult. Body yellow. Head bearing 1 + 1 white stripe with black edge in middle part, extended forward and reaching apex of genal processes, black edge incontinuity at anterior margin of vertex; vertex bearing 1 + 1 white longitudinal stripe on both sides, with black edge inside, surrounding most of lateral ocelli; outside of antennal insertion bearing 1 + 1 black edge, extended to about basal one-quarter of genal processes (Figure 7A). Genal processes slender, sometimes slightly curved inward, apex relatively acute and covered by long setae (Figure 7A). Metatibia without basal spine, apex with an open, dense crown of 7–8 strongly sclerotized apical spurs. Setae of anterior margin of forewing somewhat short (Figure 7C), range of surface spinules relatively large but away from end of each cell, range of radular spinules relatively small, that of r2 shrinking (Figure 7D). Meracanthus thin, apex somewhat acute, middle part curved downward obviously (Figure 7E).

Male terminalia: Proctiger 1.5–1.6 times as long as paramere (Figure 7F). Paramere as in Figure 7G; outer lobe irregularly, broadly triangular, blunt apically, inner surface with sclerotized band along postero-apical margin; inner lobe short and broad, triangular, strongly sclerotized apically, extending to the apical third of outer lobe. Basal segment of aedeagus mostly straight, slightly bent in the middle, relatively evenly narrowing to apex (Figure 7F). Distal segment of aedeagus as in Figure 7H,I; 1.1 times as long as basal segment; base forming inconspicuous dorsal tubercle; ventro-lateral sclerotized processes in apical quarter, relatively long and slightly curved; ventral process apical, divided just at the apex; apex irregularly rounded, hardly inflated; sclerotized end tube at aedeagal apex. Female terminalia: Dorsal surface of proctiger relatively straight, distribution area extended to proximal portion of long setae, end bearing little fine setae (Figure 7B).

Measurements (mm): Male (*n* = 2): BL 3.54–3.72; AL 4.39–4.53; HW 0.83–0.84; TW 0.67–0.68; WL 2.81–2.86. Female (*n* = 4): BL 3.84–4.02; AL 4.81–4.82; HW 0.86–0.87; TW 0.70–0.74; WL 3.08–3.17.

Fifth-instar immature unknown.

Host plant: Adults were collected on *Mucuna macrocarpa* Wall. (Fabaceae), which is a likely host.

Distribution: China (Yunnan).

#### 3.1.7. *Epipsylla nadana* Yang & Li, 1983

Figure 8 and Figure 13C,D.

*Epipsylla nadana* Yang & Li [24]: 312; Hodkinson [39] (list); Li [27] (redescription).

Material examined: Holotype: male, China, Hainan, Danzhou, Nada, 19°51′37″ N, 109°55′02″ E, 10 December 1974, Fasheng Li leg. dry-mounted; CAU. Paratypes: three males, one female, same information as holotype.

Diagnosis: Adult. Head and thorax yellowish brown and abdomen green. Head bearing 1 + 1 white stripe with black edge in middle part, extended forward but not reaching apex of genal processes; vertex bearing 1 + 1 white longitudinal stripe on both sides, with black edge inside, surrounding most of lateral ocelli; outside of antennal insertion bearing 1 + 1 black edge, extended to about basal one-third of genal processes (Figure 8A). Genal processes stout, obviously longer than median suture, apex somewhat rounded and blunt, slightly skewing outward. Metatibia without basal spine, apex with an open, dense crown of eight strongly sclerotized apical spurs. Setae of anterior margin of forewing somewhat short (Figure 8C), range of surface spinules relatively large but away from end of each cell, range of radular spinules large as well, that of r2 away from end (Figure 8D). Meracanthus stout and short, apex somewhat rounded and blunt, whole curved downward obviously (Figure 8E). Male terminalia: Proctiger 2.1–2.2 times as long as paramere (Figure 8F). Paramere as in Figure 8G; broad at base, narrower, lamellar in apical two-thirds; outer lobe irregular, lamellar, blunt apically, inner surface with sclerotized patch in apical third; inner lobe very short, subtriangular, strongly sclerotized apically, extending to the middle of outer lobe. Basal segment of aedeagus mostly straight, slightly curved basally and apically, weakly narrowing to apex (Figure 8F). Distal segment of aedeagus as in Figure 8H,I; 1.4 times as long as basal segment; base forming inconspicuous dorsal tubercle; ventro-lateral sclerotized processes in apical quarter, moderately long, only weakly curved; ventral process apical, divided near the apex into two short processes; apex irregularly rounded, weakly inflated; sclerotized end tube near aedeagal apex. Female terminalia: proctiger evenly tapered toward end, dorsal margin sloped down evenly and end hairless (Figure 8B).

Measurements (mm): Male (*n* = 2): BL 3.55–3.71; AL 4.14–4.31; HW 0.72–0.76; TW 0.64–0.66; WL 2.80–2.95. Female (*n* = 1): BL 3.36; AL 3.45; HW 0.74; TW 0.59; WL 2.69.

Fifth-instar immature and host plant unknown.

Distribution: China (Hainan).

#### 3.1.8. *Epipsylla puerariae* Yang & Li, 1983

Figure 9 and Figure 13E,F.

*Epipsylla puerariae* Yang & Li [24]: 310; Hodkinson [39] (list); Li [27] (redescription).

Material examined: Holotype: male, China, Hainan, Danzhou, Nada, 19°53′12″ N, 109°54′71″ E, 10 December 1974, Fasheng Li leg. on *Pueraria montana*; dry-mounted; CAU. Paratypes: 15 males, 13 females, 1 immature (in 75% ethanol), same information as holotype.

Diagnosis: Adult. Body green. Head bearing 1 + 1 light stripe with black edge in middle part, extended forward and wider when reaching genal processes, but not reaching apex of genal processes, outside black edge mixed with black edge of antennal insertion in apex; vertex bearing light longitudinal stripe on both sides, with black edge inside, surrounding most of lateral ocelli (Figure 9A). Genal processes relatively short and thin, straight, apex somewhat rounded and blunt, setae short (Figure 9A). Metatibia without basal spine, apex with an open, dense crown of eight strongly sclerotized apical spurs. Forewing oval, widest in middle, surface spinules present only in cell cu_2_, radular spinules of r_2_ degenerated and located at end (Figure 9D). Meracanthus relatively thin, apex somewhat acute, whole slightly curved downward (Figure 9E). Male terminalia: Proctiger 1.5 times as long as paramere (Figure 9F). Paramere as in Figure 9G; irregularly lanceolate; outer lobe irregularly triangular, subacute apically, inner surface with sclerotized area bearing a small tubercle postero-subapically; inner lobe short, subtriangular, strongly sclerotized apically, extending slightly beyond the middle of outer lobe. Basal segment of aedeagus almost straight, distinctly narrowing to apex (Figure 9F). Distal segment of aedeagus as in Figure 9H,I; 1.4 times as long as basal segment; base forming inconspicuous dorsal tubercle; ventro-lateral sclerotized processes in apical quarter, moderately long, curved; ventral process apical, inconspicuously divided near the apex; apex irregularly rounded, weakly inflated; sclerotized end tube near aedeagal apex. Female terminalia: Proctiger bearing sparse setae on both sides, apical half relatively short, apex hairless (Figure 9B).

Measurements (mm): Male (*n* = 4): BL 3.05–3.20; AL 3.26–3.46; HW 0.69–0.73; TW 0.56–0.62; WL 2.40–2.49. Female (*n* = 3): BL 3.28–3.54; AL 3.48–3.74; HW 0.72–0.75; TW 0.60–0.64; WL 2.54–2.71.

Fifth-instar immature: Abdominal margin lacking sectasetae or lanceolate setae. Outer circumanal ring comprising five rows of subrectangular pores laterally.

Host plant: *Pueraria montana* (Lour.) Merr. (Fabaceae).

Distribution: China (Hainan).

#### 3.1.9. *Epipsylla suni* Luo, Burckhardt & Cai, sp. nov.

LSIDurn:lsid:zoobank.org:act:89178311-A4D1-4ED9-88A2-68FD77952BF9

**Figure 11:**
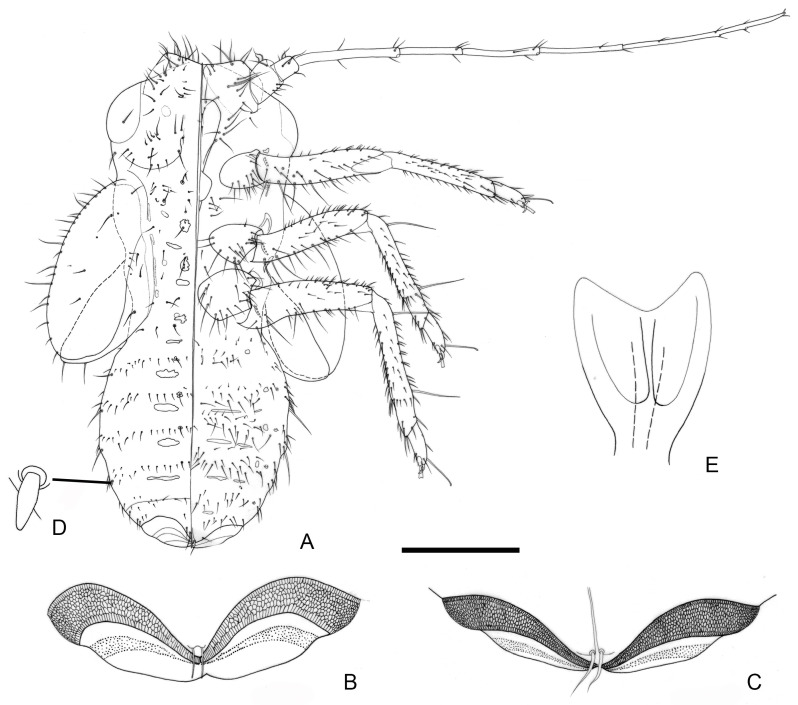
*Epipsylla suni* Luo, Burckhardt & Cai, sp. nov., fifth-instar immature. (**A**) Habitus, dorsal aspect on the left half, ventral aspect on the right half; (**B**) dorsal view of circumanal pore field; (**C**) ventral view of circumanal pore field; (**D**) blunt lanceolate seta located in margin of abdomen; (**E**) tarsal arolium. Scale bar (mm): (**A**) 0.4; (**B**) 0.12; (**C**) 0.12; (**D**) 0.03; (**E**) 0.03.

Figure 1, Figure 10, Figure 11 and Figure 13G,H.

Diagnosis: Adult. Body green overall. White longitudinal stripes of anterior part of body bearing narrow black edge on both sides (Figure 1C,D). Vertex yellowish brown in middle part and gradually darker to both sides, white longitudinal stripes of lateral margin bearing brown edge inside (Figure 10A). Genal processes slender and straight, gradually thinned to end, slightly away, apex somewhat acute (Figure 10A). Metatibia without basal spine, apex with an open, dense crown of eight strongly sclerotized apical spurs. Forewing membranous, oval, widest in apical third, anterior margin strongly arced; radular spinules thick in middle of end of cell and gathered into a long strip area, and spreading around to form small particles (Figure 10D); setae of anterior margin of forewing of different length (Figure 10C). Meracanthus relatively long, straight, apex somewhat acute, pointed to back and upward (Figure 10E). Male terminalia: Proctiger 1.4 times as long as paramere (Figure 10F). Paramere as in Figure 10G; irregularly lanceolate; outer lobe irregularly rounded, blunt apically, inner surface with sclerotized area apically; inner lobe very long, lamellar, obliquely truncate and strongly sclerotized apically, extending inner apex of outer lobe. Basal segment of aedeagus almost straight, apart from weakly curved basally; distinctly narrowing to apex (Figure 10F). Distal segment of aedeagus as in Figure 10H,I; 1.0 times as long as basal segment; base forming short dorsal process; ventro-lateral sclerotized processes in apical quarter, moderately long, hardly curved; ventral process subapical, not divided apically; apex irregularly rounded, weakly inflated; sclerotized end tube near aedeagal apex. Female terminalia: Dorsal surface of proctiger strongly rounded with a cluster of short setae on apical part (Figure 10B).

Fifth-instar immature: Abdominal margin bearing 1 + 1 lanceolate setae. Outer circumanal ring composed of each an inner and outer row of slit-like pores enclosing a band of numerous irregular pores.

Etymology: The name comes from the last name of the scholar, Ziqiang Sun, who accompanied the collector (Xinyu Luo) during the collection of the type specimens.

Type material: Holotype: male, China, Yunnan, Ruili, Nanjingli, 24°09′01″ N, 97°83′71″ E, 18 April 2014, Xinyu Luo leg. on Fabaceae sp.; dry-mounted; CAU. Paratypes: 39 males, 61 females, 12 larvae, same data as for holotype; 37 males, 44 females, 25 larvae, Yunnan, Ruili, Nanjingli, 24°09′01″ N, 97°83′71″ E, 12 April 2024, Zhixin He & Rongzhen Xu leg. on *Derris taiwaniana*; in 95% ethanol; 2 males, 2 females, same data as for holotype, NZMC; 2 males, 2 females, same data as for holotype, BMNH; 2 males, 2 females, same data as for holotype, NHMB.

Description: In addition to diagnosis, this species has the following characteristics:

Coloration: Adult. White longitudinal stripes of anterior part of body bearing narrow black edge on both sides. Pronotum yellowish brown, lateral angle of mesoscutum yellowish brown; lateral plate of each segment of thorax mostly black. Coxa of front and middle legs yellowish brown, rest of segments brown, darker in apex of tibia; coxa and trochanter of hind leg yellowish brown, femur black, tibia and tarsus yellow. Forewing somewhat yellow, veins dark brown. Abdomen completely black.

Structure: Head compressed by pronotum and slightly located below it, inclined about 80° downward with longitudinal axis of body. Surrounding part of antero-lateral angle and lateral ocelli of vertex strongly plump, anterior margin somewhat plump as a whole, boundary with antennal base blurred. Surface of base colored (with black edge) of head scaly with fine structure with relatively long setae, middle stripe area smooth and hairless. Pronotum bearing little long setae in posterior margin, plate of mesothorax and metathorax only bearing fine setae. Pronotum bearing 2 + 2 gentle small protuberances on both sides.

Male terminalia: Proctiger short and simple, not bearing posterior lobe. Sclerotized end of ejaculatory duct extended backward and slightly curved forward. Subgenital plate flat, upper margin bearing little setae, lower surface bearing dense long setae.

Female terminalia: Subgenital plate subtriangular in lateral view; upper margin bearing a row of coarsely long setae, with dense and uniform setae on both sides, gradually shorter form base to end.

Fifth-instar immature: Surface of membranous part of body covered with small granular structures, setae attached to back of body basically simple. Antennae developed, protruding forward (Figure 1E,F and Figure 11A). Antennal with seven segments, segments III and V with an apical rhinarium, respectively, and segment VII bearing two rhinaria in middle part. Ocular seta and postorbital seta long. Sclerites of dorsal side of thorax small and fragmented, central sclerites obvious, brown, and lateral sclerites relatively weak. Outer margin of hindwing pad bearing setae of varying length, anterior half bearing two small holes. Hindwing pad bearing two relatively long setae apically. Each leg relatively long, femur covered by densely short setae, tibiotarsus of middle and hind legs bearing a long seta in base of dorsal surface and proximal part. Tarsal arolium (Figure 11E) shaped as fishtail, with broad short spine and developed unguitractor; narrow as a whole, rough thickening area of palm narrow, not extended to outer margin. Dorsal sclerites of each segment of abdomen small, brown in middle part; ventral surface with fragmentary, less sclerotized sclerites. Anal plate barely sclerotized on ventral side, somewhat sclerotized on dorsal side. Anus located on the terminal of abdomen. Circumanal pore rings (Figure 11B,C) extending on both side of abdomen. Outer circumanal pore ring relatively thin in middle part and strongly widened on both sides, outermost ring as a series of long fissured pores, innermost ring as a series of longer fissured pores, and middle part as closely arranged irregular pores; inner circumanal pore ring narrow in middle and wide on both sides, and composed of small pores with irregular shape and loose arrangement. Abdomen bearing 3 + 3 setae in anterior middle of margin of dorsal surface (Figure 11A), and 1 + 1 short, unsegmented in middle, and round and blunt apically lanceolate setae (Figure 11D).

Measurements (mm): Male (*n* = 4): BL 3.09–3.38; AL 3.73–3.76; HW 0.75–0.80; TW 0.59–0.67; WL 2.52–2.66. Female (*n* = 4): BL 3.24–3.66; AL 3.52–4.32; HW 0.78–0.84; TW 0.65–0.69; WL 2.60–2.90. Fifth-instar larvae (*n* = 4): BL 1.76–1.94; AL 1.87–2.11; HW 0.72–0.76; FL 0.63–0.67.

Host plant: *Derris taiwaniana* (Hayata) Z. Q. Song (Figure 1A,B).

Distribution: China (Yunnan).

Comments: The new species is similar with *E. hainanana* in large range of surface spinules and length ratio of genal processes/vertex, but they are different in shape and position of paramere’s sclerotized area (Figure 4G versus Figure 10G) and shape of distal segment of aedeagus (Figure 4F versus Figure 10F). It is similar with *E. viridis* in anterior margin of sclerotized area of parameres visible and female terminalia, but they are different in forewing with surface spinules or not and shape of male proctiger. The type locality of new species is close to that of *E. mucunae*, but they differ from length ratio of genal processes/vertex (Figure 7A versus Figure 10A), range of surface spinules and radular spinules (Figure 7D versus Figure 10D) and shape of sclerotized area of paramere (Figure 7G versus Figure 10G) obviously. It also differs from *E. bilineata* in the distribution of surface spinules and radular spinules, and differs with *E. pulchra* in shape of male proctiger obviously.

**Figure 12:**
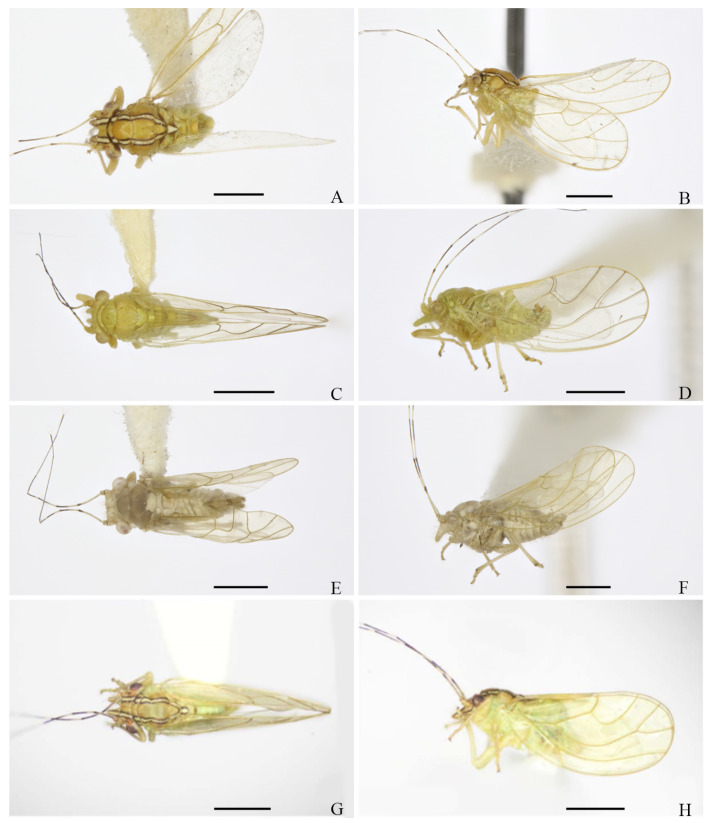
Habitus of adults. (**A**,**B**) *E. crotalariae*; (**C**,**D**) *E. guangxiana*; (**E**,**F**) *E. hainanana*; (**G**,**H**) *E. millettiae*. (**A**,**C**,**E**,**G**) Dorsal view; (**B**,**D**,**F**,**H**) lateral view. Scale bar: 1 mm.

**Figure 13:**
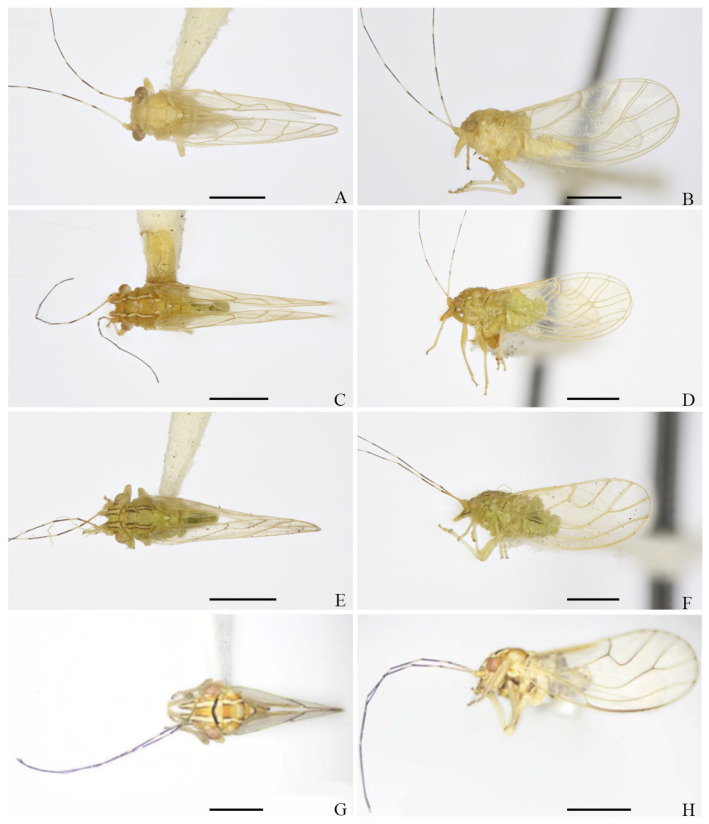
Habitus of adults. (**A**,**B**) *E. mucunae*; (**C**,**D**) *E. nadana*; (**E**,**F**) *E. puerariae*; (**G**,**H**) *E. suni*. (**A**,**C**,**E**,**G**) Dorsal dorsal view; (**B**,**D**,**F**,**H**) lateral view. Scale bars: 1 mm.

### 3.2. Key to Adults of Epipsylla spp. from Chinese Mainland

#### 3.2.1. Adults

1. Head and thorax lacking conspicuous longitudinal stripes. Sclerotized end tube of ductus ejaculatorius at mid-length of distal aedeagal segment (Figure 4I). Female terminalia subglobular (Figure 4B); dorsal margin of proctiger, in lateral view, convex·························································*E. hainanana*

- Head and thorax bearing longitudinal white stripes bordered in black (Figure 1C,D). Sclerotized end tube of ductus ejaculatorius in subapical position of distal aedeagal segment (Figure 2H and Figure 3I). Female terminalia cuneate (Figure 2B and Figure 3B); dorsal margin of proctiger, in lateral view, straight, concave or sinuate···················································································································································································································2

2. Forewing lacking surface spinules in cells r_1_, r_2_, m_1_, m_2_ and cu_1_····················································································································································3

- Forewing bearing surface spinules in cells r_1_, r_2_, m_1_, m_2_ and cu_1_·····················································································································································5

3. Outer lobe of paramere, in lateral view, symmetric, rounded apically (Figure 2F,G). Base of distal aedeagal segment with short dorsal process (Figure 2H). Apex of female proctiger, in lateral view, distinctly widened (Figure 2B)·········································································································*E. crotalariae*

- Outer lobe of paramere, in lateral view, asymmetric, blunt or subacute apically (Figure 3F,G and Figure 9F,G). Base of distal aedeagal segment with, at most, small dorsal tubercle (Figure 3I and Figure 9I). Apex of female proctiger, in lateral view, unevenly tapering to apex (Figure 3B and Figure 9B)·····························································································································································································································································4

4. Inner lobe of paramere, in lateral view, partly visible from the side, only partly hidden by outer lobe (Figure 3F,G). Dorsal margin of female proctiger strongly concave (Figure 3B); circumanal ring about half as long as proctiger········································································································*E. guangxiana*

- Inner lobe of paramere, in lateral view, not visible from the side, completely hidden by outer lobe (Figure 9F,G). Dorsal margin of female proctiger hardly concave (Figure 9B); circumanal ring about a third as long as proctiger············································································································*E. puerariae*

5. Male proctiger strongly bulged apico-posteriorly (Figure 6F). Paramere, in lateral view, slender, evenly tapering to apex (Figure 6F,G). Proximal segment of aedeagus about 2.5 times as long as wide at base (Figure 6F). Distal segment of aedeagus massive, bearing a large dorso-basal process (Figure 6I). Dorsal margin of female proctiger, in lateral view, weakly sinuate (Figure 6B)······················································································*E. millettiae*

- Male proctiger slender apico-posteriorly (Figure 5F, Figure 7F, Figure 8F and Figure 10F). Paramere, in lateral view, broader, irregularly tapering to apex (Figure 5F,G, Figure 7F,G, Figure 8F,G and Figure 10F,G). Proximal segment of aedeagus longer than 3.0 times wide at base (Figure 5F, Figure 7F, Figure 8F and Figure 10F). Distal segment of aedeagus slender, bearing, at most, a small dorso-basal tubercle (Figure 5I, Figure 7I, Figure 8I and Figure 10I). Dorsal margin of female proctiger, in lateral view, almost straight or weakly concave (Figure 5B, Figure 7B and Figure 8B) or strongly bulged (Figure 10B)··············································································································································································································································6

6. Anterior margin of inner lobe of paramere visible in lateral view. Dorsal margin of female proctiger, in lateral view, strongly bulged (Figure 10B)···············································································································································································································································*E. suni*

- Dorsal margin of female proctiger, in lateral view, almost straight or weakly concave (Figure 5B, Figure 7B and Figure 8B)················································7

7. Inner lobe of paramere completely hidden by outer lobe in lateral view (Figure 7F,G). Ventral processes of distal aedeagal segment long; apex reaching almost middle of the segment (Figure 7I). Female proctiger relatively long, blunt apically (Figure 7B)···················································*E. mucunae*

- Inner lobe of paramere not completely hidden by outer lobe in lateral view, partly visible (Figure 5F,G and Figure 8F,G). Ventral processes of distal aedeagal segment short; apex reaching apical third of the segment (Figure 7I). Female proctiger relatively short (Figure 5B), or long and pointed apically (Figure 8B)·································································································································································································································8

8. Anterior margin of inner lobe of paramere rounded (Figure 5F,G). Female proctiger relatively short, blunt apically (Figure 5B)································*E. liui*

- Anterior margin of inner lobe of paramere angular (Figure 8F,G). Female proctiger relatively long, pointed apically (Figure 8B)··························*E. nadana*

#### 3.2.2. Known Fifth-Instar Immatures

1. Abdominal margin lacking lateral sectasetae or lanceolate setae. Outer circumanal ring comprising five rows of subrectangular pores laterally. On *Pueraria montana*····················································································································································································································*E. puerariae*

- Abdominal margin bearing lateral sectasetae or lanceolate setae. Outer circumanal ring comprising more than six rows of oval pores laterally which are between the anterior and posterior margins consisting of subrectangular pores····················································································································2

2. Abdominal margin bearing a pair of sectasetae. On *Padbruggea filipes*······················································································································*E. guangxiana*

- Abdominal margin bearing a pair of lanceolate setae. On *Derris taiwaniana*·························································································································*E. suni*

### 3.3. Phylogenetic Analyses

**Figure 14:**
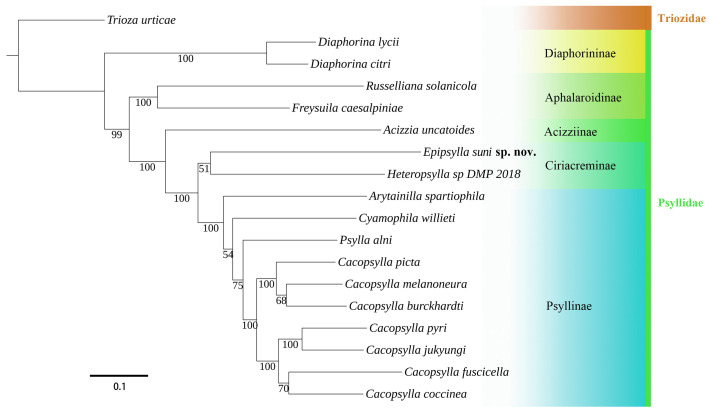
Phylogenetic relationship of Psyllidae inferred via IQ-TREE based on mitochondrial genome sequences. Numbers close to the branching points are bootstrap support values.

In the ML tree of our molecular phylogenetic analysis (Figure 14), the results support Burckhardt et al. [11]: *Epipsylla* groups with *Heteropsylla*, though this is only weakly supported. The clade *Epipsylla*–*Heteropsylla* constitutes the sister group of Psyllidae

## 4. Discussion

In the present study, we describe a new species and provide diagnoses and illustrations for eight *Epipsylla* species from the Chinese mainland. Within Ciriacreminae, *Epipsylla* species can be recognized by the combination of extremely long antennae that exceed the body length; the long conical, hairy genal processes; the metatibia lacking a genual spine and bearing a posteriorly open crown of apical spurs; the simple tubular male proctiger lacking posterior lobes; the short, complex paramere with an inner process; the relatively straight, basally massive proximal aedeagal segment; the complex distal aedeagal segment, often with a basal dorsal horn and with two latero-ventral appendices and a ventral appendix; and the relatively short cuneate female terminalia in the adults, as well as the seven-segment antennae and the posterior anus and multilayered circumanal ring in the fifth-instar immatures. Most species also display a conspicuous color feature consisting of longitudinal white stripes with black edges that extend from the apex of the genal processes to the mesoscutellum.

*Epipsylla* species are well characterized by the shape of the terminalia. While the male proctiger is relatively homogeneous in the genus, the paramere and the aedeagus display a wide range of forms. The proximal aedeagal segment has good diagnostic characteristics, which is unusual in Psyllidae. Additional diagnostic characteristics are provided by the extent of the fields of surface and radular spinules. Immatures and their diagnostic characters are largely unknown. One known character is the presence/absence of sectasetae or lanceolate setae on the abdominal margin.

As immatures are known only for some *Epipsylla* species, several of the “host” records are not confirmed by the presence of immatures [5]. Immatures and, thus, confirmed host information are available for *E. guangxiana*, *E. puerariae*, and *E. suni* but not for the other species from the Chinese mainland. Targeted field work will be necessary to fill this gap. It is also expected that additional species will be discovered.

Our phylogenetic analysis supports the placement of *Epipsylla* in *Ciriacreminae*, suggested by Burckhardt et al. [11], and is generally consistent with the results of Percy et al. [29] and Cho et al. [41]. The close relationship of *Epipsylla* with *Caradocia* and *Geijerolyma* advocated by Burckhardt et al. [11] needs further analysis.

Additional field collections and analyses in the laboratory are needed to fully understand the biodiversity, taxonomy, host plant associations, and phylogeny of this fascinating psyllid genus.

## Figures and Tables

**Table 1:** Taxa used in phylogenetic analysis.

Family	Subfamily	Species	GenBank
Triozidae		*Trioza urticae*	NC_038113.1
Psyllidae	Acizziinae	*Acizzia uncatoides*	NC_038146.1
	Aphalaroidinae	*Freysuila caesalpiniae*	NC_038135.1
		*Russelliana solanicola*	NC_038140.1
	Ciriacreminae	*Heteropsylla* sp. DMP-2018	NC_038149.1
		*Epipsyllia suni* sp. nov.	PQ645170
	Diaphorininae	*Diaphorina citri*	MF426268.1
		*Diaphorina lycii*	NC_036352.1
	Psyllinae	*Arytainilla spartiophila*	NC_038133.1
		*Cacopsylla fuscicella*	NC_080374.1
		*Cacopsylla burckhardti*	NC_069642.1
		*Cacopsylla coccinea*	NC_027087.1
		*Cacopsylla jukyungi*	NC_069847.1
		*Cacopsylla melanoneura*	OR346833.1
		*Cacopsylla picta*	OR346839.1
		*Cacopsylla pyri*	NC_038148.1
		*Cyamophila willieti*	MN364946.1
		*Psylla alni*	NC_038139.1

## Data Availability

The mitochondrial genome of *Epipsylla suni* sp. nov. has been deposited in GenBank under the accession number PQ645170.
